# Longitudinal changes of blood parameters and weight in inoperable stage III NSCLC patients treated with concurrent chemoradiotherapy followed by maintenance treatment with durvalumab

**DOI:** 10.1186/s12885-022-09395-6

**Published:** 2022-03-24

**Authors:** J. Guggenberger, S. Kenndoff, J. Taugner, L. Käsmann, B. Flörsch, C. Belka, C. Eze, F. Manapov

**Affiliations:** 1 Department of Radiation Oncology, University Hospital, LMU Munich, Munich, Germany; 2 Comprehensive Pneumology Center Munich (CPC-M), Member of the German Center for Lung Research (DZL), Munich, Germany; 3 German Cancer Consortium (DKTK), Partner Site Munich, Munich, Germany

**Keywords:** NSCLC, Durvalumab, Blood parameters, Prognostic factors, Dynamic changes

## Abstract

**Background:**

Investigating dynamic changes in blood-parameters and weight in patients with locally advanced non-small cell lung cancer (NSCLC) receiving durvalumab maintenance therapy after chemoradiotherapy (cCRT). Laboratory outcomes were determined based on the number of durvalumab administrations received.

**Methods:**

Twenty-two patients completed platinum-based cCRT followed by maintenance treatment with durvalumab. Different parameters such as hemoglobin (Hb), leukocytes, Lactate dehydrogenase (LDH), C-reactive protein (CRP), body weight and albumin were analyzed before cCRT, after cCRT, 3, 6, 9 and 12 months after starting durvalumab maintenance.

**Results:**

Sixteen (72.7%) patients were male; twelve (54.5%) and fifteen (68.2%) patients had non-squamous histology and Union for International Cancer Control (UICC) stage IIIB-C disease, respectively. Median follow-up time was 24.4 months; 12- and 18-months- progression-free and overall-survival rates were 55.0% and 45.0 as well as 90.2 and 85.0%, respectively.

During maintenance treatment Hb increased by 1.93 mg/dl (17.53%) after 9 months (*p* < 0.001) and 2.02 mg/dl (18.46%) after 12 months compared to the start of durvalumab (*p* < 0.001). LDH decreased by 29.86 U/l (− 11.74%) after 3 months (*p* = 0.022). Receipt of at least 12 cycles of durvalumab was beneficial in terms of Hb-recovery (Hb 6 months: 12.64 vs. 10.86 [mg/dl]; Hb 9 months: 13.33 vs 11.74 [mg/dl]; (*p* = 0.03)). Median weight change [kilogram (kg)] was + 6.06% (range: − 8.89 − + 18.75%) after 12 months. The number of durvalumab cycles significantly correlated with total weight gain [kg] (Spearman-Rho-correlation: *r* = 0.502*).

**Conclusion:**

In the investigated cohort, no severe hematologic toxicity occurred by laboratory blood tests within 1 year of durvalumab maintenance therapy after cCRT for unresectable stage III NSCLC. Receiving at least 12 cycles of durvalumab appears to have a significant effect on recovery of hemoglobin levels and body weight.

**Supplementary Information:**

The online version contains supplementary material available at 10.1186/s12885-022-09395-6.

## Background

Lung cancer remains the leading cause of cancer-associated death worldwide [1]. The combination of platinum-based concurrent chemoradiotherapy (cCRT) followed by maintenance therapy with the programmed cell death ligand 1 (PD-L1) inhibitor durvalumab showed a significantly improved oncological outcome than cCRT alone [2, 3]. Despite its anti-tumor activity, durvalumab maintenance therapy is associated with adverse effects [3]. Pulmonary toxicity is one well-known side effect [2–4]. General symptoms such as diarrhea or pruritus, as well as hematological toxicity have been observed [5]. Hepatitis and dermatitis have also been described as possible adverse events [5]. Endocrinological disorders such as diabetes or thyroid disease are less common [5, 6]. Hitherto, there is a paucity of data regarding hematological toxicity and especially the dynamic changes of blood parameters. C-reactive protein (CRP) levels elevated prior to treatment or during the course of treatment, as well as cancer cachexia, hypoalbuminemia, anemia and weight loss, significantly impact the prognosis of cancer patients [7–9]. Therefore, this longitudinal prospective study evaluated blood parameters of 22 patients with non-small-cell lung cancer (NSCLC) treated with cCRT and durvalumab maintenance therapy. From the start of cCRT until 1 year after the first durvalumab application, we assessed blood parameters and weight changes at regular intervals. The objective was to analyze dynamic changes during this period and possible conclusions regarding side effects and tolerability. Weight change, anemia, hypoalbuminemia, and CRP were longitudinally analyzed. Additionally, factors such as planning target volume (PTV), age, and the modified Glasgow-prognostic-Scale (mGPS) were also examined as potential influencing factors on progression.

## Methods and patients

Between September 2018 and May 2021, twenty-two (100.0%) patients underwent cCRT and consolidation durvalumab therapy. All patients had histologically or cytologically confirmed inoperable stage III NSCLC, according to UICC TNM version 8. All patients were treated with platinum-based cCRT and maintenance therapy with at least 1 cycle of durvalumab. All received platinum-based chemotherapy (cisplatin/carboplatin combined with vinorelbine/pemetrexed) and definitive radiation therapy (48–64 Gy).

Blood parameters, ECOG and weight were compared before cCRT, before receiving the first cycle of durvalumab, 3, 6, 9 and 12 months after starting durvalumab. The following blood parameters were included in the evaluation: hemoglobin [Hb], leukocytes, thrombocytes, lactate dehydrogenase (LDH), glutamic oxaloacetic transaminase (GOT)/ glutamic-pyruvate-transaminase (GPT), thyroid-stimulating hormone (TSH) and C-reactive protein (CRP), weight, and albumin. Percentage weight changes during the course of treatment were also evaluated, including CRP-albumin-ratio (CAR) and modified Glasgow-prognostic-Scale (mGPS) were determined at defined points. Reference range and units at the Ludwig-Maximilians-University (LMU) Hospital Munich were applied. The following reference values were used: Hb < 13.5 mg/dl for male patients, Hb < 11.5 mg/dl for female patients; LDH cutoff > 249 U/l; CRP < 0.5 mg/dl; leukocytes: 4.0–10.4 G/l; thrombocytes: 176–391 G/l; creatinine: 0.7–1.2 mg/dl; TSH: 0.27–4.2 μU/ml; GOT&GPT: < 50 U/l; Albumin: 3.5–5.2 g/dl. Body-Mass-Index (BMI) < 18 was defined as underweight, BMI > 18 and ≤ 25 as normal weight, BMI > 25 as overweight. To define a cut-off for CAR, we used median dichotomization. The baseline median CAR was 0.13 (range: 0.02–2.27).

Computed tomography scan [CT]/ Positron emission tomography–computed tomography [PET-CT], routine blood sample, pulmonary function testing, and clinical examinations were performed every 3 months for the first 2 years after radiotherapy, thereafter twice annually for up to 5 years, according to an in-house follow-up protocol. Contrast-enhanced brain magnetic resonance imaging (MRI), bone-scintigraphy, and bronchoscopy were only performed if clinically indicated. Local-regional recurrence (LRR) - defined as disease progression in the ipsilateral lung, bilateral mediastinum, hilum and/or supraclavicular region along with new distant metastases (DM) and brain metastasis (BM) were documented with CT, PET-CT, and MRI scans. Histological or cytological confirmation of progressive disease was not obligatory [10].

Statistical tests were performed using SPSS version 26 software (IBM, Armonk, NY) per the descriptive statistics function, paired-t-test and univariate ANOVA. Median progression-free survival [PFS] and overall survival [OS] rates were estimated using the Kaplan-Meier method with a *p*-value < 0.05 demonstrating significance.

## Results

### Patients characteristics

Patient characteristics are presented in Table 1. The median age was 67.2 years (43.6–76.8 years), 16 were male (72.7%) and nearly all patients were current or former smokers (90.9%). At baseline. Ten patients (45.5%) had chronic obstructive pulmonary disease (COPD) GOLD I-III, 45.4% had squamous cell carcinomas (SCC), whereas 45.0% had adenocarcinomas (AC). Two patients had large cell neuroendocrine tumors (LCNEC). Six patients (27.3%) had primary tumors localized in the middle or upper lobe. All 22 patients completed radiation treatment whereby 9 patients (40.9%) had a planning target volume (PTV) > 700ccm. Median PTV was 688.39 ccm [range: 204.50–1234.50 ccm]. All patients started durvalumab maintenance therapy within a median time of 4.07 weeks (2.00–14.71 weeks) after completion of cCRT. They received on average 15 cycles (range: 2–24). The occurrence of severe toxicity, poor compliance or confirmed progression were reasons for treatment discontinuation.

**Table 1:** Patient characteristics

	Number of patients (%)
Age	
≤ 65 years	9 (41)
> 65 years	13 (59)
Sex	
Female	6 (27)
Male	16 (73)
Eastern Co-operative Oncology Group (ECOG) performance status (PS)	
0	11 (50)
1	9 (41)
2	2 (9)
UICC stage	
III	22 (100)
T descriptor	
1–2	6 (27)
3–4	16 (73)
N descriptor	
0–1	5 (23)
2–3	17 (77)
Histology	
Squamous cell carcinoma (SCC)	10 (45)
Adenocarcinoma (AC)	10 (45)
Large cell neuroendocrine cancer (LCNEC)	2 (9)
Tobacco consumption (PY)	
0	2 (9)
20–40	8 (36)
> 40	12 (55)
Durvalumab completion rate	
50% (12 or more cycles)	15 (68)
100% (24 cycles)	8 (36)
Reason for treatment discontinuation	
Progress	8 (50)
Toxicity	5 (31)
Compliance	1 (6)
Induction chemotherapy	7 (32)
Concurrent chemotherapy	21 (95)
PTV > 700 ccm	9 (41)
PTV < 700 ccm	13 (59)

Eighteen patients (81.8%) presented with anemia prior to cCRT and 13 patients (59.1%) had initially elevated LDH values. For five patients (22.7%), an initial mGPS > 1 was calculated, 11 patients (50.0%) had elevated CAR before cCRT. Eight patients (36.4%) completed all 24 cycles of durvalumab, 15 patients (68.2%) received at least half of the planned number of cycles (≥12 cycles durvalumab). Median number of cycles applied was 15 (range 2–24). Reasons for interruption were tumor progression (*n* = 8), severe lung or skin toxicity (*n* = 5) or compliance issues (*n* = 1). At the cutoff date (May 26, 2021), 11 patients (50%) had disease progression of which six had died. Median follow-up time was 24.4 months; 12- and 18-month PFS was 55.0 and 45.0%, respectively; 12-month-overall-survival was 90.2%, 18-month-overall-survival was 85.0%.

### Hemoglobin

**Fig. 1:**
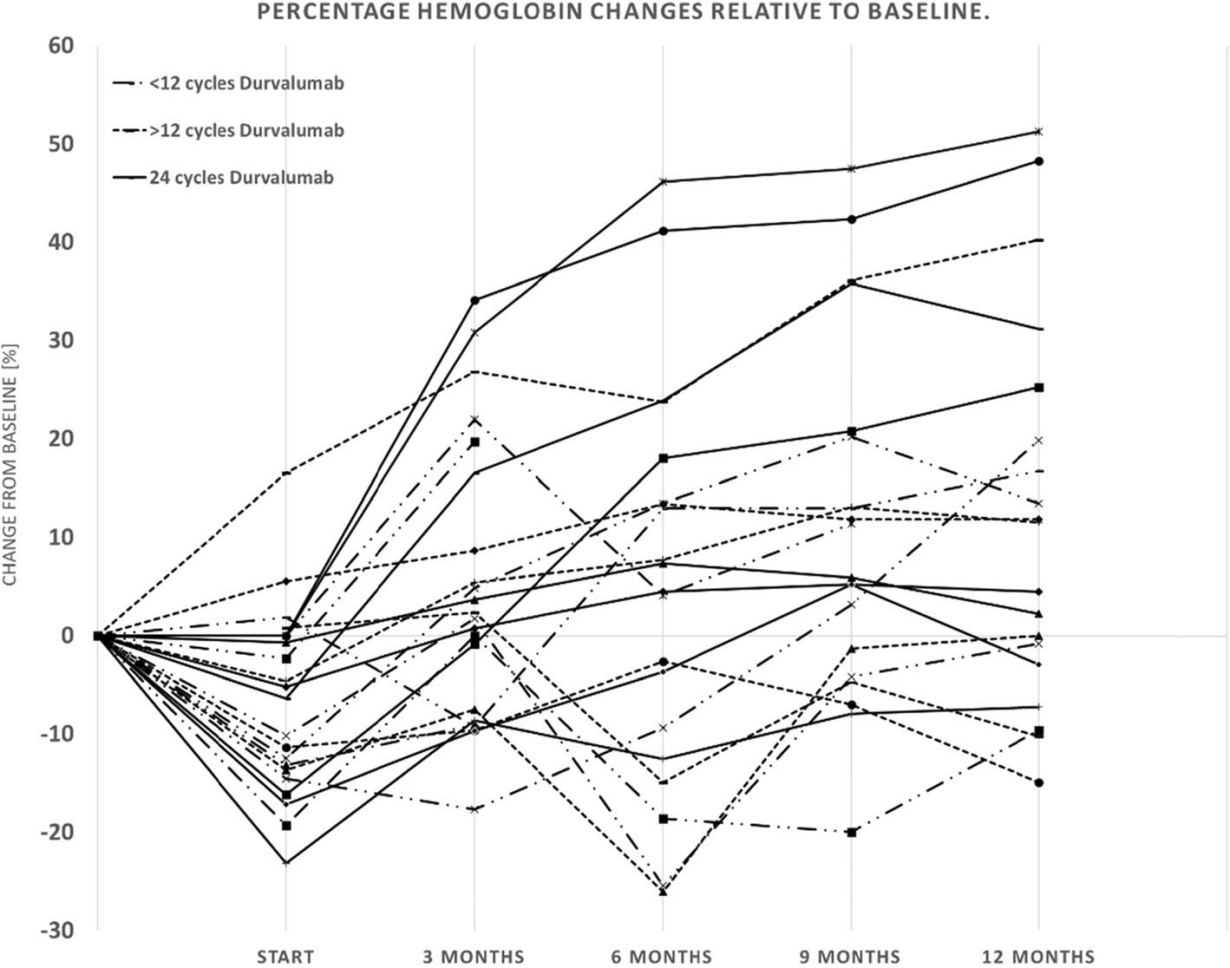
Percentage hemoglobin changes relative to baseline. Legend: Percent change in hemoglobin [%] of each patient in three categorized groups - the solid line for patients receiving 24 cycles of durvalumab, the dotted line for patients receiving at least 12 cycles of durvalumab, and the broken line for patients receiving less than 12 cycles of durvalumab

Mean Hb values were 11.9 g/dl (*n* = 22, range 7.8–15.1 g/dl, standard deviation (SD) = ±1.9); 11.1 g/dl (*n* = 22, range 7.8–13.5 g/dl, SD = ±1.7); 12.4 g/dl (*n* = 22, range 7.9–15.2 g/dl, SD = ±1.9); 12.2 g/dl (*n* = 20, range 8.7–14.6 g/dl, SD = ±1.6); 12.9 g/dl (*n* = 20, range 9.9–14.8 g/dl, SD = ±1.5); 13.0 g/dl (*n* = 19, range 9.7–14.6 g/dl, SD = ±1.3). See figure (Fig. 1).

By means of a paired-t-test, the initial Hb values were compared with the values during the course of treatment. Mean Hb-values after completion of cCRT were significantly lower than prior to cCRT (− 7.22%, *p* = 0.003). Mean Hb at 3 months increased by 1.30 mg/dl (11.76%) compared to the start of durvalumab (*p* < 0.001) and mean Hb levels at 6 months increased by 1.18 mg/dl (10.81%; *p* = 0.006). After 9 months it increased by 1.93 mg/dl (17.53%) compared to the start of durvalumab (*p* = < 0.001). At the end of observation after 12 months, there was an increase in mean Hb of 1.12 mg/dl compared to the baseline (9.46%; *p* = 0.020), and an increase of 2.02 mg/dl (18.46%) compared to the start of durvalumab (*p* = < 0.001).

There was no significant difference in Hb development between patients older or younger than 65 years at diagnosis (*p* values: 0.1, 0.14, 0.08, 0.06, 0.06). The occurrence of a progression after cCRT did not significantly affect the recovery of the Hb levels (*p* values: 0.3, 0.47, 0.69, 0.78, 0.89).

Furthermore, we performed univariate ANOVA analyses for testing if Hb levels correlated with pre-defined groups: neither PTV > 700 ccm (*p* values: 0.3; 0.21; 0.1; 0.45; 0.32; 0.45) nor initial mGPS ≥1 (*p* values: 0.99; 0.99; 0.27; 0.41; 0.87; 0.43) nor increased baseline LDH (*p* values: 0.29; 0.58; 0.16; 0.25; 0.09; 0.7) significantly correlated with dynamic changes of Hb. Patients who received at least 12 cycles of durvalumab had significantly higher Hb values after 6, 12.64 mg/dl vs. 10.86 mg/dl (*p* = 0.03) and 9 months, 13.33 mg/dl vs. 11.74 mg/dl (*p* = 0.03). There was no significant difference after 12 months (*p* = 0.23).

For an overview, refer to Tables 1, 2 and 3 in the supplementary file.

### Lactate dehydrogenase (LDH)

**Fig. 2:**
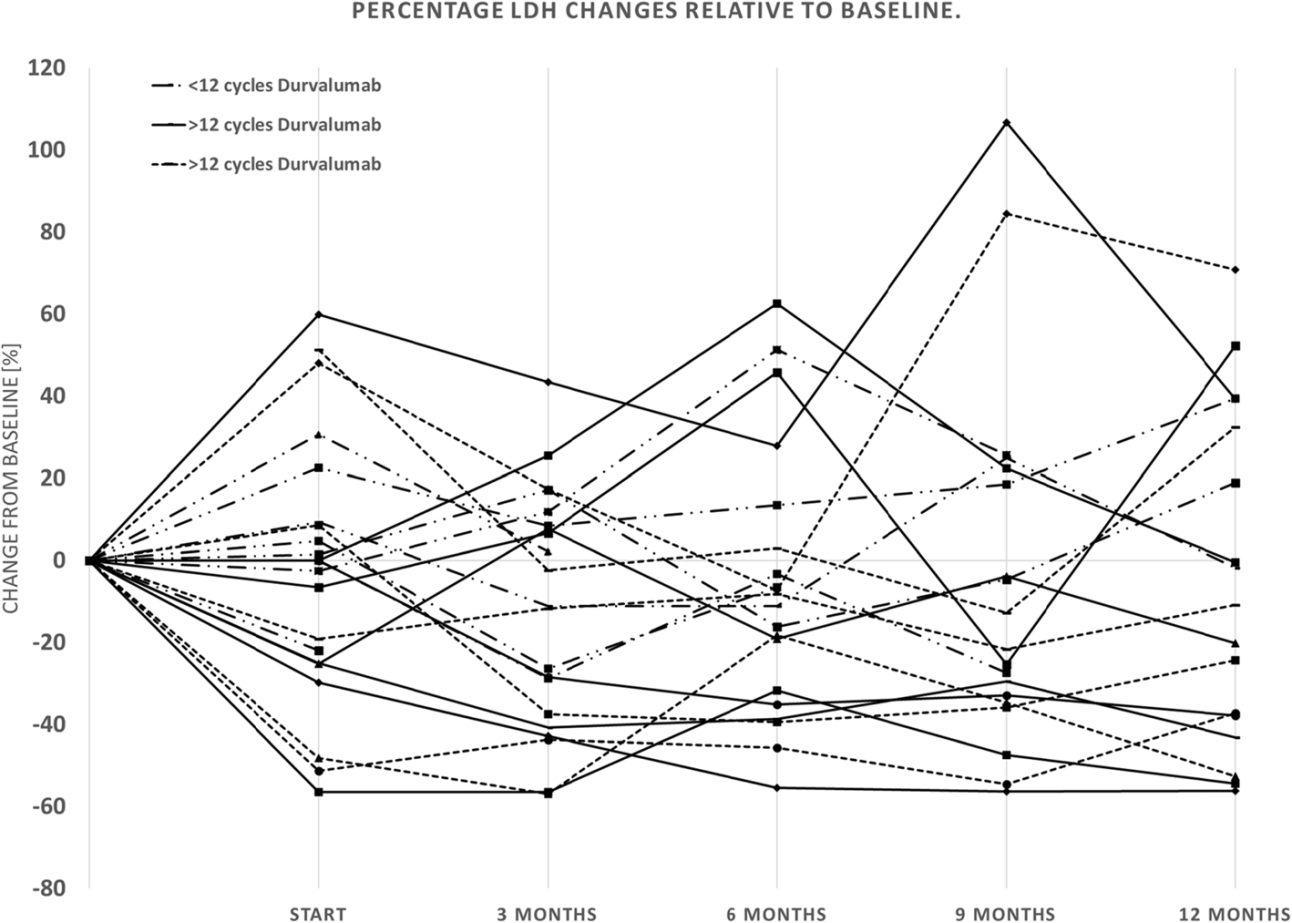
Percentage LDH changes relative to baseline. Legend: Percent change in LDH [%] of each patient in three categorized groups - the solid line for patients receiving 24 cycles of durvalumab, the dotted line for patients receiving at least 12 cycles of durvalumab, and the broken line for patients receiving less than 12 cycles of durvalumab

Mean LDH values were: 276.5 U/l (*n* = 22, range 178.0–444.0 U/l, SD = ±74.1); 254.6 U/l (*n* = 22, range 149.0–425.0 U/l, SD = ±64.0); 224.5 U/l (*n* = 21, range 170.0–308.0 U/l, SD = ±40.3); 246.2 U/l (*n* = 20, range 166.0–379.0 U/l, SD = ±68.5); 238.9 U/l (*n* = 19, range 139.0–404.0 U/l, SD = ±69.8); 240.6 U/l (*n* = 17, range 163.0–396.0 U/l, SD = ±73.4) for the respective examination times described above. See Fig. 2.

Using a paired-t-test analogous to the methodology used above, we compared initial LDH values with the values during the treatment course. Mean LDH-values after completion of cCRT were not significantly lower than prior to cCRT, (− 11.18%; *p* = 0.15). Mean LDH at 3 months decreased by 29.86 U/l (− 11.74%) compared to durvalumab initiation (*p* = 0.02) and mean LDH at 6 months decreased by 8.75 U/l (3.43%; *p* = 0.34). After 9 months it decreased by 11.95 U/l (4.76%) compared to durvalumab initiation (*p* = 0.26). At the end of the observation period after 12 months, there was a decrease in mean LDH of 36.47 U/l (− 13.16%) compared to baseline (*p* = 0.24) and a decreased mean LDH of 8.53 U/l (3.42%) compared to durvalumab initiation (*p* = 0.3).

LDH values after 9 and 12 months were significantly higher in the group older than 65 years (*p* < 0.05), no significant difference in LDH development from starting durvalumab up to 6 months later was found between those groups (*p* values: 0.06, 0.08, 0.54). The occurrence of a progression after cCRT did not significantly affect the LDH levels (*p* values: 0.83, 0.96, 0.11, 0.18, 0.34, 0.81).

The dynamic changes of LDH values were also examined for a possible correlation within the previously described groups. Neither PTV > 700 ccm (*p* values: 0.44; 0.91; 0.23; 0.16; 0.85; 0.41) nor initial mGPS ≥1 (*p* values: 0.57; 0.75; 0.28; 0.71; 0.82; 0.07) was associated with dynamic changes of LDH. There was also no significant difference between patients who completed or discontinued therapy (*p*-values: 0.44; 0.46; 0.28; 0.78; 0.96; 0.43).

For an overview, see Tables 1, 2 and 3 in the supplementary file.

### CRP

Based on all values measured in the group, CRP > 5 mg/dl was defined as “elevated CRP”. One patient (4.5%, CRP = 5.8 mg/dl) had elevated CRP before starting durvalumab, 2 patients (9.1% CRP = 8.0 and 10.5 mg/dl) after 3 months, 2 patient (9.1%, CRP = 18.7 and 6.0 g/dl) after 6 months, 1 patient (4.5%, CRP = 9.0 mg/dl) after 9 months and 12 months after the start of consolidation therapy, no patient with an increased CRP value of > 5 mg/dl could be identified (see Table 2).

**Table 2:** CRP levels

	Before cCRT	Before Durvalumab	3-month follow-up	6-month follow-up	9-month follow-up	12-month follow-up
Patient values	22	22	22	19	19	18
CRP ≤5	20 (91%)	21 (95%)	19 (86%)	17 (89%)	18 (95%)	18 (100%)
CRP > 5	2 (9%)	1 (5%)	3 (14%)	2 (11%)	1 (5%)	0

### Weight changes and albumin

According to the WHO definition, the BMI of the patients before cCRT was calculated. Twelve patients (54.5%) were normal weight (range: 20–24) and 10 patients (45.5%) were overweight (range: 26–35). After the last follow-up (12 months), 8 patients (47.0%) were normal weight (range: 18–25), 9 patients (53%) were overweight (range: 26–37). Overall, median total weight change was 6.1% (range: − 8.89- + 18.75%). Four patients suffered weight loss (median weight loss was 5.2%, range: − 3.2% - -8.89%); 10 patients gained weight (median weight gain was 10.6%, range: 1.8–18.8%). For a detailed presentation of the individual changes after 12-month follow-up, see Table 3. In addition, there was a moderate correlation between the number of durvalumab cycles given and weight gain [kg], (Spearman-Rho-correlation, *r* = 0.502*). Administration of at least 12 cycles of durvalumab (*r* = 0.346) as well as 24 cycles (*r* = 0.410) correlated with an increase in weight (kg) at the end of observation.

**Table 3:** Mean values of thrombocytes, leucocytes, GOT, GPT, TSH and albumin at the following time points: before cCRT; before Durvalumab; at 3-, 6-, 9- and 12-month follow-up. We also calculated weight changes as a percentage of baseline at each time point

	Reference value	Baseline	Start Durvalumab	3-month follow-up	6-month follow-up	9-month follow-up	12-month follow-up
Thrombocytes [G/l]	176–391	286.91	247.68	254.41	267.00	252.70	279.63
Leukocytes [G/l]	4–10.4	7.70	5.83	6.35	6.81	6.81	7.30
GOT [U/l]	> 49	25.82	26.59	27.95	27.79	27.16	30.17
GPT [U/l]	> 49	27.64	28.64	28.50	29.84	31.26	36.56
TSH [μU/ml]	0.27–4.2	1.35	1.34	1.64	3.09	1.86	2.05
Albumin [g/dl]	3,5 - 5,2	4.01	4.09	4.10	4.25	4.18	4.20
Weight change [%]	–	–	- 2	0	+ 2	+ 4	+ 3

Overall, mean albumin values remained stable and within the reference range (see Tables 1 and 3 in the supplementary file). At baseline, one patient (4.5%) had hypoalbuminemia after cCRT and up to 3 months after starting durvalumab, no hypoalbuminemia occurred. During further observation, one patient (4.5%) had hypoalbuminemia at 6 and 9 months, respectively. Hyperalbuminemia was not observed at any time during the entire follow-up.

### Liver function, kidney function and thyroid levels

Within the entire observation period, values for TSH and creatinine remained within the reference range, see Table 1 in the supplementary file. Univariate ANOVA analyses did not show any significant difference between the age groups (> 65 years) (*p* values: 0.4; 0.72; 0.95; 0.32; 0.66; 0.69) or patients with/without progression (*p* values: 0.94; 0.89; 0.62; 0.23; 0.57; 0.48), summarized in Table 3 in the supplementary file.

Two (9.0%) patients suffered grade 3 asymptomatic thyroiditis after cCRT and was detected only by elevated TSH (TSH > 8μU/ml). After substitution therapy, TSH levels returned to normal during the course of treatment.

Mean GOT and GPT values as well as TSH, thrombocytes and leukocytes remained within the reference range. For more details, see Tables 1 and 3 in the supplementary file.

### Correlation with a historical cohort

In order to compare our contemporary cohort with a cohort of stage IIIA-C NSCLC patients treated with concurrent cCRT/RT without immunotherapy, we collected identical parameters in a cohort of 16 patients with similar data collection at various time points and enrolled in a prospective observational study.

One-factor ANOVA analyses between the groups showed no significant difference, see Table 4.

**Table 4:** ANOVA analysis comparing patients with and without recieving durvalumab maintenance therapy regarding LDH, hemoglobin, leukocytes, thrombocytes, GOT, GPT and albumin

Parameter	Baseline	Begin Durvalumab	3 months FU	6 months FU	9 months FU	12 months FU
	*p*-value	*p*-value	*p*-value	*p*-value	*p*-value	*p*-value
LDH	0.784	0.440	0.349	0.589	0.965	0.537
Hemoglobin	0.774	0.530	0.196	0.222	**0.015**	0.115
Leukocytes	0.528	0.413	0.882	0.778	0.336	0.762
Thrombocytes	0.294	0.835	0.749	0.929	0.757	0.122
GOT	0.724	0.053	0.421	0.883	0.949	0.827
GPT	0.291	0.096	0.060	0.048	0.199	0.284
Albumin	0.538	0.116	0.967	0.304	0.164	0.195

## Discussion

As consolidation treatment following cCRT in inoperable stage III NSCLC, durvalumab has shown promising results by boosting the body’s anti-tumor immunity and has significantly improved PFS and OS rates [3]. Documentation of safety and long-term side effects is an important tool to further improve maintenance therapy and is prognostically invaluable. Therefore, we analyzed the longitudinal changes of blood parameters and weight of our patient cohort.

No severe hematological toxicity was observed during durvalumab maintenance. Subsequent anemia following CRT and at the start of durvalumab treatment, regressed throughout the course of durvalumab maintenance. As expected, there was a significant decrease in Hb value after completed cCRT (*p* < 0.05). Compared to the Hb levels at the beginning of durvalumab maintenance therapy, there was a significant increase after 3 months (*p* < 0.05), 6 months (0.01) and 9 months (*p* < 0.05). At the end of FU after 12 months, anemia had recovered significantly (*p* < 0.05). Interestingly, the final Hb value even showed a significant increase compared to the baseline Hb before cCRT (*p* = 0.02). Hemoglobin recovery at 6 and 9 months appeared to be associated with at least 12 durvalumab cycles (both *p* = 0.03). However, this suggestion is most likely biased by disease progression and salvage treatment in patients who progressed on durvalumab. Despite this, the impact of durvalumab on Hb levels should be further investigated in a larger prospective cohort. Leukocyte and platelet count also remained within the reference ranges during the whole observation period. These findings suggest that durvalumab maintenance therapy did not cause any hematological events within the investigated cohort. Consistent with the TATTON study [11], which describes, among other things, a very low rate of cytopenia with immunotherapy, the dynamics of this cohort show promising Hb, leukocyte, and platelet development.

The course of LDH, which is a ubiquitous and non-specific marker of cell damage, also suggests good tolerability of durvalumab. The LDH values show interesting dynamics as there is a statistically significant decrease 3 months after the start of maintenance therapy when comparing the mean values of LDH (*p* = 0.022). Advanced age over 65 years is associated with higher LDH levels at 9 and 12 months in this cohort (*p* = 0.02, *p* = 0.01). This may be an incidental finding but should also be critically assessed by long-term observation. This suggests a good physical tolerability of durvalumab. After a total of 6 and 9 months, the LDH value remains stable, the slightly but non-significant increase after 12 months (*p* = 0.41) must be evaluated. The stable values for CRP, GOT/GPT, and creatinine over time also show that hepatotoxic or renal side effects were unlikely within the investigated cohort. No subjective symptoms were reported by the patients in this regard either. These results support the findings of other studies and underline the good hematological tolerability of durvalumab [12–14]. As described in the results section, two patients (9.0%) showed asymptomatic hypothyroidism, which is most likely explained by radiation-induced thyroiditis. Fortunately, substitution therapy led to normalization of thyroid levels. Thyroid disorders are frequently described as side effects of durvalumab therapy [12, 13, 15, 16]. Therefore, the monitoring of blood parameters during durvalumab maintenance therapy showed a definite therapeutic benefit for these patients, as the disorder could be detected and corrected at an early stage. On average, the dynamics of weight change shows stabilization and partial improvement of weight loss. The significant positive correlation between the number of durvalumab cycles and weight gain (*r* = 0,502*) at the end of observation suggests a positive impact of durvalumab therapy on body weight in investigated cohort. Weight gain under CRT alone leads to better OS and prolonged distant metastasis-free survival) [17]. Thus, it can be hypothesized that successful maintenance therapy can act as a protective factor regarding tumor cachexia, which should be investigated in further studies.

Albumin, weight changes and anemia are indicators of the nutritional status and potential tumor cachexia [6, 7, 18]. The dynamic changes in the described cohort show a stable course, which suggests a continuous improvement in quality of life. As we demonstrated, patients recovered from initial anemia and presented with decent ECOG status and stable BMI 12 months after initiation of durvalumab therapy. Encouragingly, marked hypoalbuminemia did not occur in any case.

Known predictive factors such as the mGPS, PTV > 700 ccm, elevated LDH levels or a high CAR were not associated with PFS or OS within the observation period [11–16, 19–21]. We acknowledge the limitations of the study, particularly both the small sample size and associated impact on the statistical analysis as well as the relatively short follow-up time.

In summary, the cohort showed significant recovery of anemia to baseline Hb and stable dynamics of LDH levels. Comparison with a historical cohort without immunotherapy also showed no significant differences. Thus, it can be assumed that durvalumab maintenance therapy is not associated with significant changes in terms of clinical laboratory parameters. The absence of critical CRP increase or evidence of significant tumor cachexia as well as the stable persistence of the other described parameters underline the tolerability of durvalumab maintenance therapy. Studies with a longer FU and larger sample size are pertinent for long-term assessment of laboratory parameters and more robust statistical analyses to confirm the findings of this study.

## Conclusion

Within 1 year of durvalumab consolidation therapy after cCRT for inoperable stage III NSCLC, no severe hematologic or organ toxicity was observed by laboratory blood tests. Patients who received at least 12 cycles of durvalumab showed significant recovery in hemoglobin levels and body weight. Prognostic unfavourable markers such as critical CRP progression or tumor cachexia were not observed.

## Supplementary Information

**Additional file 1: Table 1.** Mean value of all analyzed parameters, including range and standard deviation [SD] at all time points (Baseline, begin durvalumab, 3 months follow up [FU], 6 months FU, 9 months FU, 12 months FU).

**Additional file 2: Table 2.** Summary of paired t-tests comparing values between the defined times of LDH and hemoglobin.

**Additional file 3: Table 3.** ANOVA analyses on LDH, hemoglobin and TSH for comparison between defined groups.

## Data Availability

Data was generated at LMU Hospital Großhadern, Munich. Derived data supporting the findings of this study are available from the corresponding author on request.

## Abbreviations

AC: Adenocarcinoma
BM: Brain metastasis
BMI: Body-Mass-Index
CAR: CRP-albumin-ratio
cCRT: Chemoradiotherapy
COPD: Chronic obstructive pulmonary disease
CRP: C-reactive protein
CT: Computed tomography
DM: Distant metastases
FU: Follow up
GOT: Glutamic oxaloacetic transaminase
GPT: Glutamic-pyruvate-transaminase
Hb: Hemoglobin
kg: Kilogram
LCNEC: Large cell neuroendocrine tumors
LDH: Lactate dehydrogenase
LMU: Ludwig-Maximilians-University
LRR: Local-regional recurrence
mGPS: Glasgow-prognostic-Scale
MRI: Magnetic resonance imaging
NSCLC: Non-small cell lung cancer
OS: Overall survival
PD-L1: Programmed cell death ligand 1
PET-CT: Positron emission tomography–computed tomography
PFS: Progression-free survival
PTV: Planning target volume
SCC: Squamous cell carcinoma
SD: Standard deviation
TSH: Thyroid-stimulating hormone
UICC: Union for International Cancer Control
