# Supplementary material for: Longitudinal changes of blood parameters and weight in inoperable stage III NSCLC patients treated with concurrent chemoradiotherapy followed by maintenance treatment with durvalumab
Source: BMC Cancer. 2022 Mar 24;22:317. doi: 10.1186/s12885-022-09395-6 (PMC8944024; doi:10.1186/s12885-022-09395-6)
Supplement: Supplementary file 1 — Additional file 1: Table 1. Mean value of all analyzed parameters, including range and standard deviation [SD] at all time points (Baseline, begin durvalumab, 3 months follow up [FU], 6 months FU, 9 months FU, 12 months FU). [file 12885_2022_9395_MOESM1_ESM.docx]

**Supplements**

**Table 1:** Mean value of all analyzed parameters, including range and standard deviation [SD] at all time points (Baseline, begin durvalumab, 3 months follow up [FU], 6 months FU, 9 months FU, 12 months FU).

| Parameter | Baseline | | | Begin Durvalumab | | | 3 months FU | | | 6 months FU | | | 9 months FU | | | 12 months FU | | |
| --- | --- | --- | --- | --- | --- | --- | --- | --- | --- | --- | --- | --- | --- | --- | --- | --- | --- | --- |
|  | Value | range | SD | Value | range | SD | Value | range | SD | Value | range | SD | Value | range | SD | Value | range | SD |
| LDH [U/l] | 276.5 | 178.0-444.0 | ±74.1 | 254.6 | 149.0- 425.0 | ±64.0 | 224.5 | 170.0- 308.0 | ±40.3 | 246.2 | 166.0- 379.0 | ±68.5 | 238.9 | 139.0- 404.0 | ±69.8 | 240.6 | 163.0- 396.0 | ±73.4 |
| Hemoglobin [g/dl] | 11.9 | 7.8- 15.1 | ±1.9 | 11.1 | 7.8- 13.5 | ±1.7 | 12.4 | 7.9- 15.2 | ±1.9 | 12.2 | 8.7- 14.6 | ±1.6 | 12.9 | 9.9- 14.8 | ±1.5 | 13.0 | 9.7- 14.6 | ±1.3 |
| Leukocytes [G/l] | 7.70 | 2.79- 12.50 | ±2.7 | 5.83 | 2.4-10.7 | ±2.1 | 6.35 | 3.7- 9.8 | ±2.0 | 6.81 | 3.5- 10.4 | ±1.8 | 6.81 | 4.1- 11.4 | ±2.0 | 7.30 | 3.3- 12.8 | ±2.8 |
| Thrombocytes [G/l] | 286.91 | 192.0- 568.0 | ±48.8 | 247.68 | 138.0- 359.0 | ±58.1 | 254.41 | 138.0- 377.0 | ±65.4 | 267.00 | 117.0- 588.0 | ±100.7 | 252.70 | 172.0- 411.0 | ±64.5 | 279.63 | 150.0- 500.0 | ±103.6 |
| GOT [U/l] | 25.82 | 12.0- 51.0 | ±9.7 | 26.59 | 12.0- 74.0 | ±14.0 | 27.95 | 14.0- 64.0 | ±11.0 | 27.79 | 12.0- 44.0 | ±9.0 | 27.16 | 12.0- 42.0 | ±7.0 | 30.17 | 15.0- 125.0 | ±24.4 |
| GPT [U/l] | 27.64 | 8.0- 78.0 | ±17.5 | 28.64 | 9.0- 133.0 | ±28.4 | 28.50 | 5.0- 77.0 | ±17.1 | 29.84 | 8.0- 49.0 | ±13.2 | 31.26 | 8.0- 61.0 | ±16.4 | 36.56 | 7.0- 220.0 | ±47.3 |
| Albumin [g/dl] | 4.01 | 2.9- 4.6 | ±0.4 | 4.09 | 3.4- 4.9 | ±0.5 | 4.10 | 3.2- 4.8 | ±0.5 | 4.25 | 3.4- 4.9 | ±0.4 | 4.18 | 3.2- 4.8 | ±0.4 | 4.20 | 3.4- 4.6 | ±0.3 |
| Creatinine | 0.96 | 0.7- 1.4 | ±0.2 | 1.02 | 0.7- 1.3 | ±0.2 | 1.04 | 0.6- 1.6 | ±0.3 | 1.10 | 0.7- 1.7 | ±0.3 | 1.07 | 0.7- 1.4 | ±1.9 | 1.10 | 0.7- 1.8 | ±0.3 |
| TSH [µU/ml] | 1.35 | 0.2- 4.1 | ±0.9 | 1.34 | 0.1- 4.1 | ±1.0 | 1.64 | 0.01- 4.3 | ±1.3 | 3.09 | 0.2- 20.3 | ±4.3 | 1.86 | 0.3- 4.3 | ±1.3 | 2.05 | 0.4- 7.7 | ±2.1 |
