# Supplementary material for: Longitudinal changes of blood parameters and weight in inoperable stage III NSCLC patients treated with concurrent chemoradiotherapy followed by maintenance treatment with durvalumab
Source: BMC Cancer. 2022 Mar 24;22:317. doi: 10.1186/s12885-022-09395-6 (PMC8944024; doi:10.1186/s12885-022-09395-6)
Supplement: Supplementary file 2 — Additional file 2: Table 2. Summary of paired t-tests comparing values between the defined times of LDH and hemoglobin. [file 12885_2022_9395_MOESM2_ESM.docx]

**Supplements**

**Table 2:** Summary of paired t-tests comparing values between the defined times of LDH and hemoglobin.

| Parameter | before cCRT -> begin Durvalumab | | begin Durvalumab -> 3 months FU | | begin Durvalumab -> 6 months FU | | begin Durvalumab -> 9 months FU | | begin Durvalumab -> 12 months FU | | Baseline -> 12 months FU | | |
| --- | --- | --- | --- | --- | --- | --- | --- | --- | --- | --- | --- | --- | --- |
|  | δ | p-value | δ | p-value | δ | p-value | δ | p-value | δ | p-value | δ | p-value |  |
| LDH [U/l] | -21.91 | 0.964 | -29.86 | **0.020** | -8.75 | 0.776 | -11.94 | 0.146 | -8.53 | 0.017 | -36.47 | 0.242 |  |
| Hemoglobin [%] | -7.22 | **0.003** | 11.76 | **<0.001** | 10.81 | **0.006** | 17.53 | **<0.001** | 18.46 | **<0.001** | 9.46 | **0.020** |  |
