# Supplementary material for: Longitudinal changes of blood parameters and weight in inoperable stage III NSCLC patients treated with concurrent chemoradiotherapy followed by maintenance treatment with durvalumab
Source: BMC Cancer. 2022 Mar 24;22:317. doi: 10.1186/s12885-022-09395-6 (PMC8944024; doi:10.1186/s12885-022-09395-6)
Supplement: Supplementary file 3 — Additional file 3: Table 3. ANOVA analyses on LDH, hemoglobin and TSH for comparison between defined groups. [file 12885_2022_9395_MOESM3_ESM.docx]

**Supplements**

**Table 3:** ANOVA analyses on LDH, hemoglobin and TSH for comparison between defined groups.

| Parameter | Correlated groups | Baseline | Begin Durvalumab | 3 months FU | 6 months FU | 9 months FU | 12 months FU |
| --- | --- | --- | --- | --- | --- | --- | --- |
|  |  | p-value | p-value | p-value | p-value | p-value | p-value |
| LDH | <65 / >65 | 0.91 | 0.06 | 0.08 | 0.54 | **0.01** | **0.01** |
|  | PTV >700 ccm | 0.44 | 0.91 | 0.23 | 0.16 | 0.85 | 0.41 |
|  | Initial mGPS ≥1 | 0.57 | 0.75 | 0.28 | 0.71 | 0.82 | 0.07 |
|  | Completed/ discontinued therapy | 0.44 | 0.46 | 0.28 | 0.78 | 0.96 | 0.43 |
|  | Progression | 0.83 | 0.96 | 0.11 | 0.18 | 0.34 | 0.81 |
| Hemoglobin | <65 / >65 | 0.10 | **0.003** | 0.14 | 0.08 | 0.06 | 0.06 |
|  | PTV >700 ccm | 0.30 | 0.21 | 0.10 | 0.45 | 0.32 | 0.45 |
|  | Initial mGPS ≥1 | 0.99 | 0.99 | 0.27 | 0.41 | 0.87 | 0.43 |
|  | Increased LDH | 0.29 | 0.58 | 0.16 | 0.25 | 0.09 | 0.70 |
|  | Completed/ discontinued therapy | 0.92 | 0.86 | 0.08 | **0.03** | **0.03** | 0.23 |
| TSH | <65 / >65 | 0.40 | 0.72 | 0.95 | 0.32 | 0.66 | 0.69 |
|  | Progression | 0.94 | 0.89 | 0.62 | 0.23 | 0.57 | 0.48 |
